# Intracardiac electrophysiology to characterize susceptibility to ventricular arrhythmias in murine models

**DOI:** 10.3389/fphys.2024.1326663

**Published:** 2024-01-23

**Authors:** Marine C. Ferrand, Gauthier Giordano, Nathalie Mougenot, Pierre-Léo Laporte, Nicolas Vignier, Arnaud Leclerc, Vincent Algalarrondo, Fabrice Extramiana, Flavien Charpentier, Nathalie Neyroud

**Affiliations:** ^1^ Sorbonne Université, Inserm, Research Unit on Cardiovascular and Metabolic Diseases, UMRS-1166, Paris, France; ^2^ Competence Center for Hereditary or Rare Heart Diseases, Centre Hospitalier Régional Universitaire de Nancy, Vandœuvre-lès-Nancy, France; ^3^ Sorbonne Université, Plateforme PECMV, UMS-28, Paris, France; ^4^ Reference Center for Inherited Arrhythmic Syndromes, Hôpital Bichat, APHP, Université de Paris Cité, Paris, France; ^5^ Sorbonne Université, Inserm, UMRS-974, Center of Research in Myology, Institute of Myology, Paris, France; ^6^ Nantes Université, CNRS, INSERM, L’Institut du Thorax, Nantes, France

**Keywords:** programmed electrical stimulation, ventricular arrhythmias, electrocardiography, electrophysiology, channelopathies, cardiomyopathies, animal model, protocol

## Abstract

**Introduction:** Sudden cardiac death (SCD) and ventricular fibrillation are rare but severe complications of many cardiovascular diseases and represent a major health issue worldwide. Although the primary causes are often acute or chronic coronary diseases, genetic conditions, such as inherited channelopathies or non-ischemic cardiomyopathies are leading causes of SCD among the young. However, relevant experimental models to study the underlying mechanisms of arrhythmias and develop new therapies are still needed. The number of genetically engineered mouse models with cardiac phenotype is growing, making electrophysiological studies in mice essential tools to study arrhythmogenicity and arrhythmia mechanisms and to test novel treatments. Recently, intracardiac catheterization via the jugular vein was described to induce and record ventricular arrhythmias in living anesthetized mice. Several strategies have been reported, developed in healthy wild-type animals and based on aggressive right ventricular stimulation.

**Methods:** Here, we report a protocol based on programmed electrical stimulation (PES) performed in clinical practice in patients with cardiac rhythm disorders, adapted to two transgenic mice models of arrhythmia - Brugada syndrome and cardiolaminopathy.

**Results:** We show that this progressive protocol, based on a limited number of right ventricular extrastimuli, enables to reveal different rhythmic phenotypes between control and diseased mice. In this study, we provide detailed information on PES in mice, including catheter positioning, stimulation protocols, intracardiac and surface ECG interpretation and we reveal a higher susceptibility of two mouse lines to experience triggered ventricular arrhythmias, when compared to control mice.

**Discussion:** Overall, this technique allows to characterize arrhythmias and provides results in phenotyping 2 arrhythmogenic-disease murine models.

## 1 Introduction

The burden of cardiovascular death remains a leading cause of premature death in most countries. Sudden cardiac death (SCD) due to ventricular arrhythmias (VAs) is one of the first causes of global mortality and accounts for approximately 50% of all cardiovascular deaths (Amini et al., 2021). SCD and VAs are severe complications of several cardiovascular diseases. Although the main origin is acute or chronic coronary artery disease (Fox et al., 2004), genetic conditions, such as inherited channelopathies or non-ischemic inherited cardiomyopathies are leading causes among the young (Ackerman et al., 2016).

The improvement of genome sequencing has helped to better classify and treat inherited arrhythmia syndromes and to assess their specific arrhythmic risk. This led the European Society of Cardiology to upgrade the recommendation of genetic testing to evaluate the risk of SCD in several conditions (Zeppenfeld et al., 2022). According to these recommendations, several non-invasive and invasive tests may be useful for risk stratification. As a diagnostic and prognostic tool, programmed electrical stimulation (PES) may guide therapies in some genetic conditions, such as Brugada syndrome (Sroubek et al., 2016; Gaita et al., 2023) and non-ischemic dilated cardiomyopathy (Dilaveris et al., 2017).

Animal models, particularly genetically modified mice, have played a crucial role in elucidating the pathophysiology of channelopathies and cardiomyopathies (Clauss et al., 2019). Such models have been used to study functional consequences of specific genetic mutations, as well as the interactions between mutations and indirect factors, such as tissue remodeling (fibrosis), or environmental factors (exercise, stress), on cardiac electrical activity. However, arrhythmias only develop at tissue or organ level. Accordingly, animal models should reproduce the arrhythmic phenotype observed in diseased patients if they are to be used to understand arrhythmia mechanisms and/or test potential treatments. Electrophysiological studies in transgenic mice have provided major insights in the field of hereditary arrhythmias (Huang, 2017). Nevertheless, since the occurrence of spontaneous ventricular arrhythmias is very low in mice (Nerbonne, 2014), PES is necessary to induce arrhythmic events and is a relevant tool to evaluate the arrhythmogenicity of a model, to study arrhythmia underlying mechanisms and to test potential therapeutic interventions such as drugs or gene therapy.

Over time, PES techniques have been adapted to the murine context and refined from open-chest to transesophageal and transjugular approaches (Berul et al., 1996; Hagendorff et al., 1999; Li and Wehrens, 2010; Clasen et al., 2018). Very recently, 2 intracardiac stimulation protocols dedicated to mice were elaborated and detailed, both of them aiming at triggering arrhythmias in wild-type (WT) mice, by using aggressive “burst” stimulation programs (Favere et al., 2022; Hennis et al., 2022). Considering that in human clinical practice, increasing the number of extrastimuli during PES decreases diagnostic and prognostic accuracy (Brugada et al., 1984), we assumed that developing less aggressive protocols, based on clinical experience, to trigger ventricular arrhythmias in mice would avoid inducing arrhythmias in normal animals and thus help differentiating normal from diseased ventricles. Here, we describe the surgical procedure to perform PES in mice, we detail all needed materials and we propose adapted protocols, based on the repetition of 2–4 extrastimuli, that allowed us to unveil the higher susceptibility to develop VAs of a Brugada-like mouse model and a model of arrhythmogenic cardiomyopathy, without any pharmacological provocation. The aim of this study is to provide the research community working in the field of cardiac arrhythmias with reproducible, ready-to-use and standardized protocols to perform PES in mice for the purpose of characterizing arrhythmias in transgenic murine models of cardiac diseases by comparison with WT mice.

## 2 Materials and equipment

### 2.1 Mice

All animal experiments complied with the Guide for the Care and Use of Laboratory Animals, according to the Directive 2010/63/EU of the European Parliament, and were approved by the local committee of animal care (Agreement B751320). The protocols presented here can be performed in mice from 2 months of age (18–25 g). In this study, experiments have been operated in male WT, *Scn5a* ^+/−^ or *Lmna* ^H222P/H222P^ C57BL/6J mice (Vignier et al., 2019), C57BL/6 being one of the most commonly used mouse line in research laboratories. Mice were 2–4 months of age in the *Scn5a* ^+/−^ group and 2–6 months in the *Lmna* ^H222P/H222P^ group. They were kept in standard cages on a standard diet and water *ad libitum* at a constant temperature of 22°C in a 12:12-hour controlled light/dark cycle. In this study, all 3 types of mice (WT, *Scn5a* ^+/−^ and *Lmna* ^H222P/H222P^) were randomly stimulated by an experimenter who had no knowledge of their genotype.

Disruption of the mouse cardiac sodium channel gene, *Scn5a*, causes intra-uterine lethality in homozygotes with severe defects in ventricular morphogenesis whereas heterozygotes show normal survival. In ventricular myocytes isolated from adult *Scn5a* ^+/−^ mice, sodium conductance was reduced by approximately 50%. As a result, *Scn5a* ^+/−^ hearts reveal several defects including impaired atrioventricular conduction, delayed intramyocardial conduction, increased ventricular refractoriness, and ventricular tachycardia with characteristics of reentrant excitation, as shown in Langendorff-perfused mouse ventricular preparations (Papadatos et al., 2002).

The *Lmna* ^H222P/H222P^ mouse model was created by introducing a mutation identified in a family with autosomal dominant Emery-Dreifuss muscular dystrophy, one of the laminopathies presenting an arrhythmogenic cardiomyopathy phenotype. At adulthood, male homozygous mice display reduced locomotion activity with abnormal stiff walking posture and they all die by 9 months of age. As for cardiac phenotype, they develop chamber dilatation, hypokinesia and conduction defects revealed by an increased PR interval, indicating atrio-ventricular conduction defects and an increased QRS interval, a parameter of intraventricular conduction (Arimura et al., 2005).

### 2.2 Surgical kit

• Heating pad (Minerve Equipement Vétérinaire, France)
• Fine forceps n°12060-01 (Fine Science Tools, CA, USA)
• Sharp scissors n°03-336-115K (Allgaier Germany Stainless, Germany)
• Micro-scissors n°15000-00 (Fine Science Tools, CA, USA)
• Braided silk suture (Corza Medical, MA, USA)
• Endotracheal tube (Harvard Apparatus, MS, USA)

### 2.3 ECG monitoring materials

• Subdermal 29 G needle electrodes (ADINSTRUMENTS, New Zealand)
• ECG cable: cable with five extensions for 4 recording electrodes and 1 reference electrode (emka TECHNOLOGIES, France)
• IOX analog-digital converter (emka TECHNOLOGIES, France)
• IOX2 acquisition software (emka TECHNOLOGIES, France)
• ecgAUTO analysis software (emka TECHNOLOGIES, France)

### 2.4 Intracardiac recording and stimulation materials

• Octopolar catheter 1.1 F with 8 rings configurable in “recording” or “stimulating” mode (Transonic, NY, USA)
• Extension Cable for Octapolar ECG Catheter (emka TECHNOLOGIES, France)
• 8-channel general-purpose stimulus generator with filter amplifier providing current and voltage-driven electrical stimulation (Multi Channel Systems, Germany)
• MC_Stimulus II software (Multi Channel Systems, Germany)
• Amplifier with 1 input for 7 lead ECG recording and 1 bridge amplifier channel for direct USB link to IOX acquisition software (emka TECHNOLOGIES, France)
• Tergazyme (Alconox, NY, USA)

### 2.5 Drugs

• Etomidate 12 mg/kg (2 mg/mL, Piramal Critical Care, PA, USA)
• Xylovet 93 mg/kg (injectable solution of Lidocaine 21.33 mg/mL) Ceva Santé Animale, France)
• Euthasol vet. 150 mg/kg (injection solution, 400 mg/mL pentobarbital sodium, Dechra, UK)

## 3 Methods

### 3.1 Surface ECG

Before performing PES, baseline surface ECG is recorded in each anesthetized mouse using 29 G needle electrodes connected to the IOX analog-digital converter for 3 min. The anesthetic etomidate was chosen for its minimal effects on heart rate (Leoni et al., 2010; Gergye et al., 2020).

**FIGURE 1:**
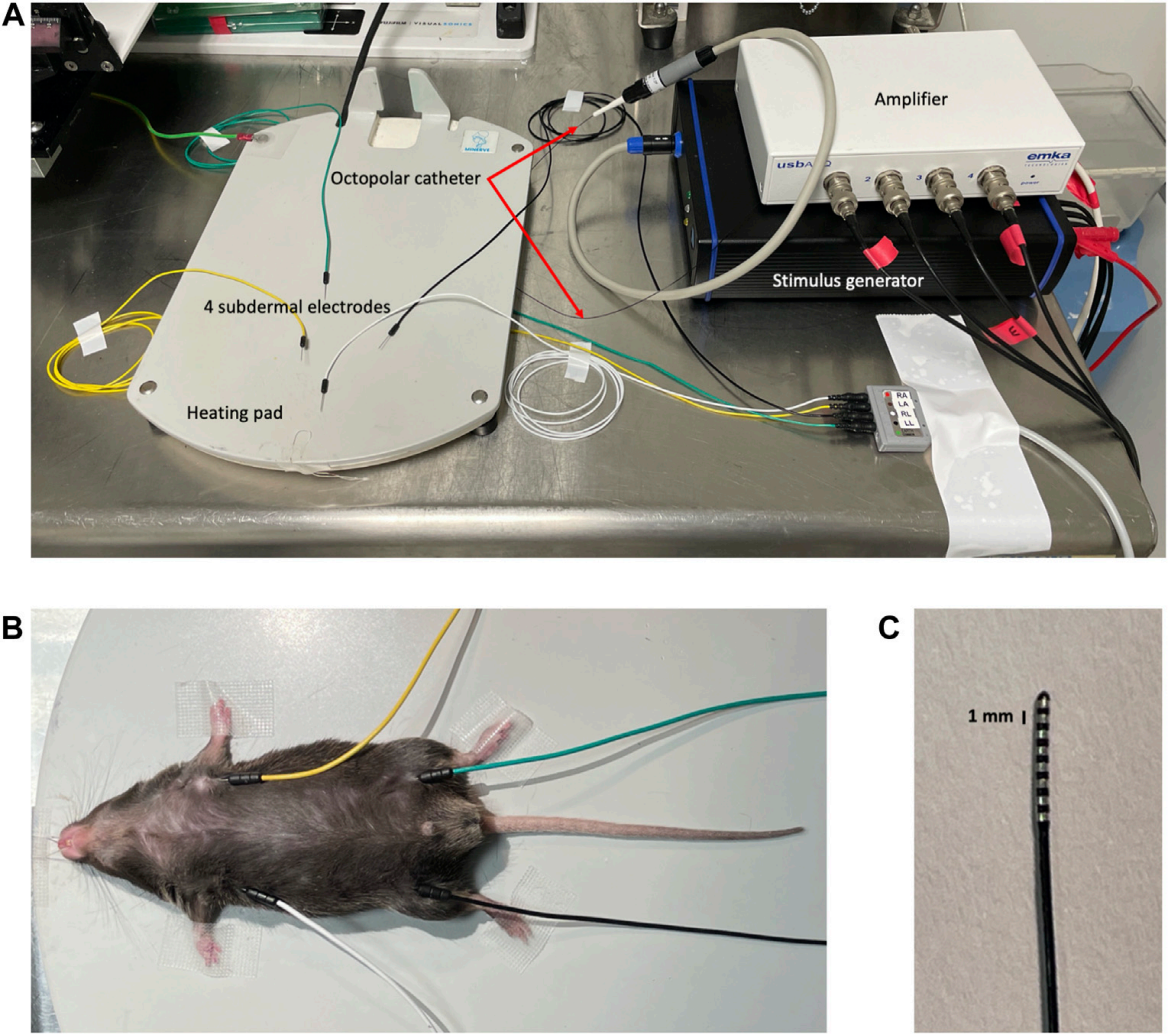
PES setup. **(A)** Overview of the experimental setup composed of heating pad, subdermal 29 G needle electrodes, stimulus generator, amplifier and ECG cable. **(B)** Mouse under etomidate anaesthesia with subdermal 29 G needle electrodes inserted under the skin of all four extremities to record surface ECG using the IOX2 acquisition software. **(C)** Octopolar catheter tip showing the 8 rings, 4 of which are used for stimulating or recording intracardiac electrogram.

1. Set up a heating pad, four subdermal needle electrodes, ECG cable, IOX analog-digital converter and the octopolar catheter connected to stimulus generator on a laboratory bench (Figure 1A).
2. Weigh the mouse.
3. Induce anesthesia by intraperitoneal injection of etomidate (150 μL for a 18–25 g mouse). As soon as the mouse is unconscious, pursue with a subcutaneous injection of Xylocaine (100 μL for a 18–25 g mouse) in the surgical area for local anesthesia.
4. Wait until the mouse is properly anesthetized by verifying the absence of tail or toe pinch reflexes
5. Place the mouse in supine position on the heating pad at 37°C, orientated with animal’s head close to the experimenter and attach the 4 limbs to the table.
6. Insert needle electrodes subcutaneously into both front limbs and the left hind limb to record surface ECG according to Einthoven (leads I, II and III). Ground the signal by placing a fourth needle electrode into the right hind limb (Figure 1B).
7. Open IOX2 software and start recording baseline surface ECG for 3 min.

### 3.2 Surgical procedure

Before surgical procedure for intracardiac stimulation, moisten the octopolar catheter (Figure 1C) with 0.9% NaCl sterile solution to lubricate its tip. Open IOX2 and MC_Stimulus II software; place all the 4 most distal rings of the catheter in recording mode and adjust IOX2 setting so that surface ECG is displayed in channels 1 and 2 and intracardiac electrogram in channels 3 to 6.

**FIGURE 2:**
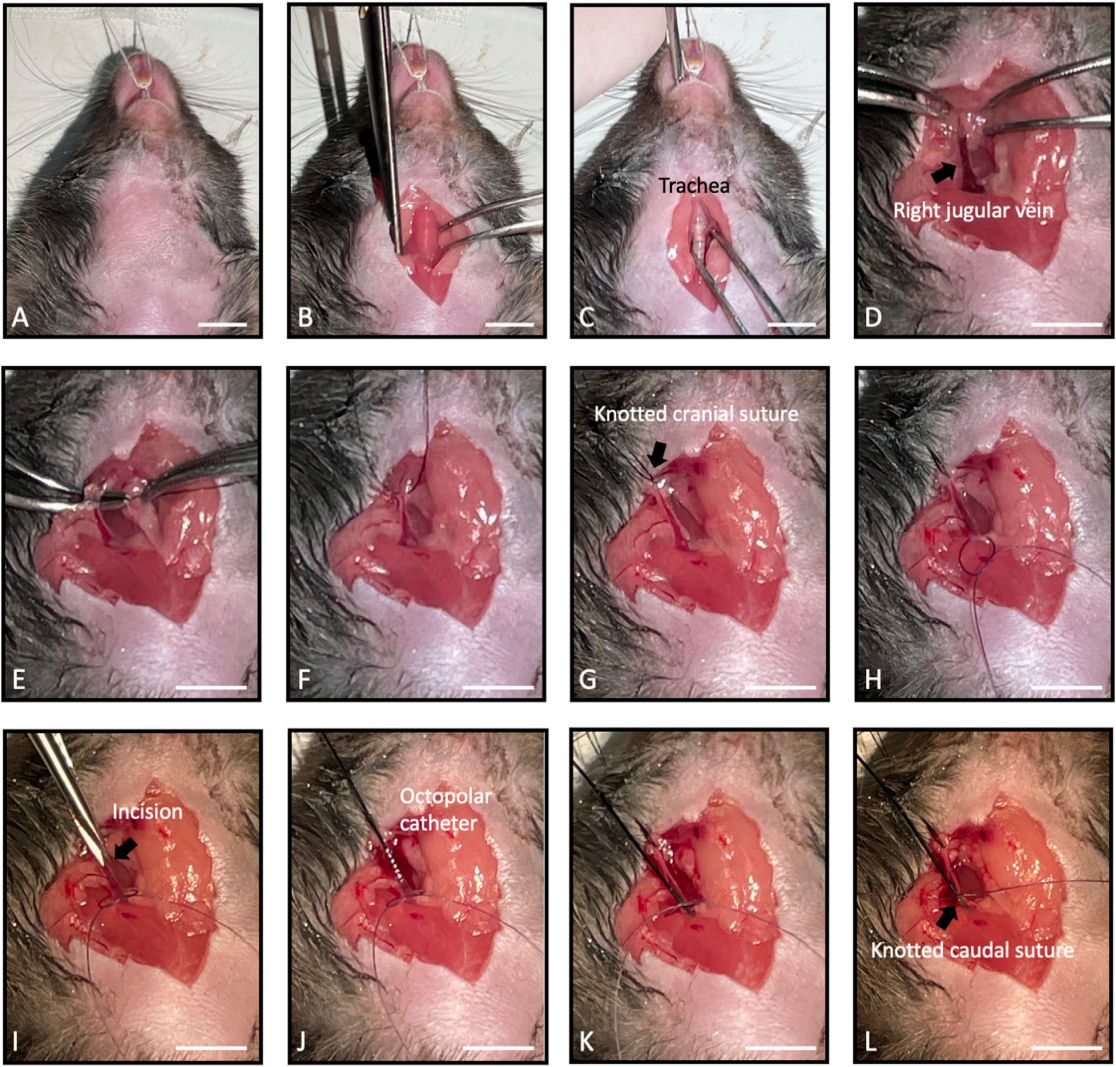
Key steps in the surgical procedure and catheter positioning. **(A)** Remove hair from the surgical area. **(B)** Incise the skin around the animal’s throat. **(C)** Intubate the mouse with an endotracheal tube. **(D)** Expose the right jugular vein (indicated by the black arrow). **(E,F)** Place a suture wire around the cranial side of the vein. **(G)** Close the suture and pull the wire in cranial direction. The black arrow indicates the suture knot **(H)** Place a second suture wire with a loose knot around the caudal side of the vein. **(I)** Make an incision in the upper surface of the jugular vein. The black arrow indicates the opening **(J)** Insert the catheter into the opening. **(K)** Slide the catheter slowly toward the heart. **(L)** Secure the second suture to maintain the catheter for the duration of recordings. Scale bars: 10 mm.

Remove animal hair from the surgical area using depilatory cream (Figure 2A); use sharp scissors to incise the skin around the animal’s throat (Figure 2B) and intubate the mouse with an endotracheal cannula to help maintain spontaneous breathing without using mechanical ventilation system (Figure 2C).

1. Use sharp scissors and fine forceps to remove skin from below the chin toward the transversal pectoral muscles on the right side of the animal.
2. Expose the right jugular vein by pushing back the salivary glands and fatty tissues. Dissect surrounding tissues (Figure 2D).
3. Place a suture wire around the cranial side of the vein (Figures 2E, F). Close this suture to stop blood flow and pull the wire in cranial direction (Figure 2G). Tape it to the table to maintain tension on the vein and facilitate catheter insertion. Place a second suture wire around the caudal side of the vein and place a loose knot on this suture (Figure 2H).
4. Use micro-scissors to make an incision in the upper surface of the jugular vein (Figure 2I).
5. Insert the catheter into the opening (Figure 2J).
6. Slide the catheter slowly toward the heart (Figure 2K) while monitoring its position based on the intracardiac electrogram signal on the computer screen.

**FIGURE 3:**
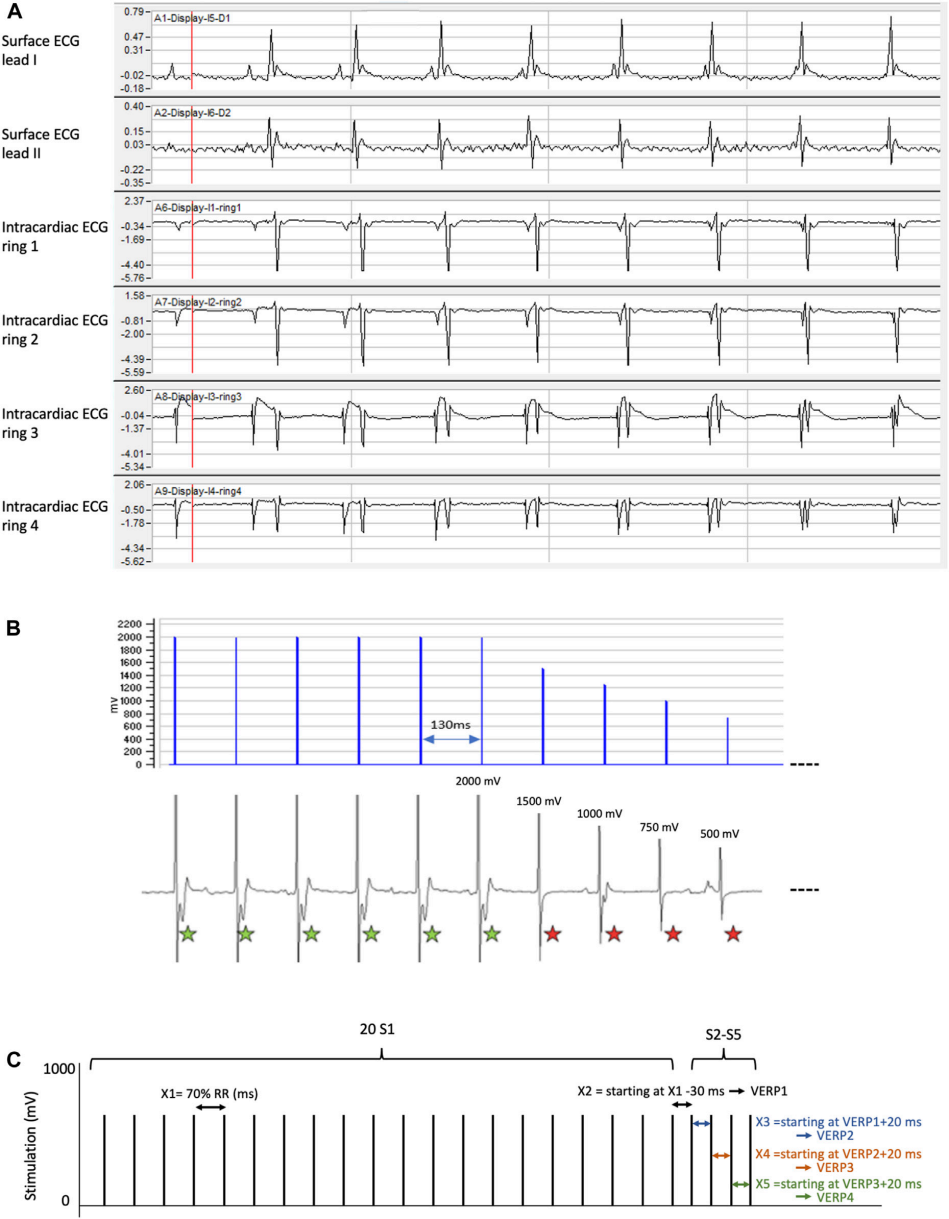
Acquisition and stimulation parameters set-up. **(A)** Intracardiac and surface ECG signals. IOX2 acquisition software is set to display surface ECG leads I and II in the 2 first channels and unipolar endocardial electrogram recorded from the catheter electrodes 1, 2, 3 and 4 respectively in the 4 following channels. In our system, ring 1 is the most distal electrode of the octopolar catheter. Correct position of the catheter within the right ventricle can be assessed by following the changes in the morphology of recorded intracardiac complexes (prominent ventricular electrical activity in ring 1 vs*.* prominent atrio-ventricular electrical activity in ring 4). **(B)** Determination of stimulation threshold. Upper panel: schematic representation of the protocol designed to assess the stimulation threshold of each mouse. Lower panel: corresponding surface ECG recording. In this example, the threshold is measured at 2,000 mV. The green star indicates a ventricular capture and the red star, an absence of capture. **(C)** Diagram of a standard ventricular arrhythmia induction protocol. First, 20 S1 stimuli are delivered (interval X1 = 70% basal RR), followed by the delivery of S2 starting at X1 -30 ms and decreasing in 2 ms steps to measure VERP1. S2 is followed by S3 (interval X3 starting at VERP1 +20 ms). Extrastimuli are delivered in a similar way until S5, delivered to obtain VERP4.

Verify the correct location of the catheter by comparing intracardiac and surface ECG traces: atrial signal should be synchronized with the P wave and ventricular signal with the QRS. Amplitude and morphology of atrial and ventricular intracardiac electrograms also help to follow the catheter electrodes sliding from atria to ventricle (Figure 3A). In this study, we only used the 4 most distal rings on the catheter for ventricular stimulation and recording.

7. Once the catheter is located in the desired position (right ventricle apex), secure the second suture to maintain the catheter for the duration of recordings (Figure 2L).

We chose to focus this methodological description of PES to ventricular arrhythmia models and to detail how we adapted human protocols to the murine context. Nevertheless, the system described here can be used to perform PES for atrial testing with protocols published elsewhere (Mesirca et al., 2016; Clasen et al., 2018; Favere et al., 2022; Hennis et al., 2022).

### 3.3 Programmed electrical stimulation for ventricular testing

Stimulation protocols are designed and applied using MC Stimulus II software. Stimulation impulses are rectangular with a pulse duration of 2 ms. Pacing is bipolar, i.e., between two adjacent catheter electrodes, the two most distal ones in this case. Before applying any protocol, it is required to switch all catheter channels to recording mode before placing the two most distal channels 1 and 2 to stimulation mode. Also, in order to facilitate ECG analysis, it is important to save, close and open a new file between each protocol.

#### 3.3.1 Determination of the stimulation threshold

In all the following protocols, the pacing cycle length (S1–S1) corresponds to 70% of the animal spontaneous RR interval recorded during baseline surface ECG. This cycle length has been empirically established to systematically capture the heart. It was 120 ms for the vast majority of mice. Stimulation is performed at an intensity of twice the capture threshold to insure capture throughout all ventricular protocols. The stimulation threshold is established by pacing the heart using decreasing voltage amplitude from 2000 to 150 mV (Figure 3B). The lowest voltage triggering a capture is considered as the stimulation threshold.

#### 3.3.2 Determination of the ventricular effective refractory period

**FIGURE 4:**
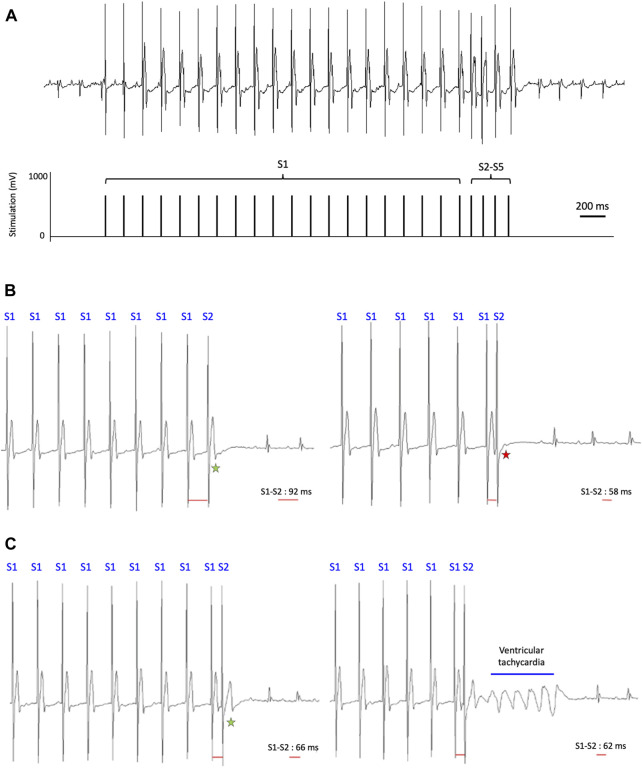
PES protocols for ventricular arrythmia induction. **(A)** PES protocol consisting of the repetition of 20 S1 stimuli at a cycle length of 70% of the mouse RR interval, followed by 4 extrastimuli S2–S5 that are coupled in decrements of 2-ms intervals from 10 ms above VERP to 30 ms below VERP. **(B)** Determination of the VERP. Stimulation trains of 20 S1 stimuli are applied, followed by delivery of a single extrastimulus S2 at a cycle length starting 30 ms below S1–S1 interval and decreases by 2-ms steps. Left: S1–S2 interval (92 ms in this example) can be adjusted to each mouse to ensure capture (indicated by the green star). Right: The protocol can be stopped as soon as S2 extrastimuli do not capture (indicated by the red star). The VERP corresponds to the longest S1–S2 cycle length which does not trigger a heartbeat (58 ms in this example). **(C)** PES protocol to induce ventricular arrhythmia. Left: As S1–S2 cycle length approaches the mouse VERP, the induced heartbeat (indicated by the green star) widens, suggesting local disturbances in conduction velocity. Right: VT triggered by shortening of the S1–S2 cycle length close to the mouse VERP.

As previously introduced, the aim of this methodological manuscript is to design and detail PES protocols inspired from human clinical practice. Thus, we propose here protocols based on the repetition of 2–4 extrastimuli, i.e., S2 to S5, coming after repetition of 20 S1 stimuli (Figure 4A).

To determine the ventricular effective refractory period (VERP), we deliver a train of S1 stimulations followed by a single premature extrastimulus S2, with an S1–S2 cycle length shortening by 2-ms steps until the ventricular capture is lost (Figure 4B).

1. Switch the two most distal catheter channels to stimulation mode
2. Deliver stimulation trains of 20 S1 stimuli with a cycle length of 70% of the RR recorded on baseline ECG, followed by delivery of a single extrastimulus S2. S1–S2 cycle length starts 30 ms below S1–S1 interval and decreases by 2-ms steps. S1–S2 interval can be adjusted to each mouse or model to ensure capture (Figure 4B left panel).
3. The protocol can be stopped as soon as the S2 extrastimulus does not capture twice in succession (Figure 4B right panel). The VERP corresponds to the longest S1–S2 cycle length, which does not trigger a heartbeat, i.e., capture.

#### 3.3.3 Arrhythmia induction protocol

To study the susceptibility to VAs induced by PES in a Brugada-like mouse model and a model of cardiolaminopathy, we applied protocols repeating 2 to 4 extrastimuli with a shortening cycle length, named S2–S5 protocols. This approach allows establishing 4 consecutive VERP values and, in so doing, to assess mice arrhythmia susceptibility by challenging the ventricular tissue as we stimulate at a cycle length close to its local refractory period. Figure 4C shows how the ventricular depolarization induced by the S2 extrastimulus slows (wider QRS and delayed S2-QRS interval; left panel) because of relative refractory period. Conduction slowing associated with spatial ventricular repolarization heterogeneity pave the way for reentrant phenomenon (right panel).

1. Switch the two most distal catheter channels to stimulation mode
2. Deliver stimulation trains of 20 S1 stimuli with the cycle length established in the previous protocol, followed by delivery of S2 with S1–S2 cycle length of VERP + 10 ms, followed by a single S3 stimulus. S1–S2 cycle length has been empirically chosen 10 ms above VERP to ensure capture of ventricular tissue. S2–S3 cycle length starts 20 ms above VERP and decreases by 2-ms steps. VERP2 corresponds to the longest S2–S3 cycle length without S3 capturing the ventricle.
3. Deliver stimulation trains of 20 S1 stimuli, followed by delivery of S2 with S1–S2 cycle length of VERP + 10 ms, followed by delivery of S3 with S2–S3 cycle length of VERP2 + 10 ms, followed by a single S4 stimulus. S3–S4 cycle length starts 20 ms above VERP2 and decreases by 2-ms steps. VERP3 corresponds to the longest S3-S4 cycle length without S4 capturing the ventricle.
4. Deliver stimulation trains of 20 S1 stimuli, followed by delivery of S2 with S1–S2 cycle length of VERP + 10 ms, followed by delivery of S3 with S2–S3 cycle length of VERP2 + 10 ms, followed by delivery of S4 with S3–S4 cycle length of VERP3 + 10 ms, followed by a single S5 stimulus. S4-S5 cycle length starts 20 ms above VERP3 and decreases by 2-ms steps. VERP4 corresponds to the longest S4-S5 cycle length without S5 capturing the ventricle.

#### 3.3.4 Arrhythmia-induction “burst” protocol

In order to challenge our approach of progressive protocols and compare it with protocols detailed in control mice, we also applied a “burst” protocol to our group of WT mice. This protocol consists in the delivery of 20 stimuli at an aggressive cycle length of 20 ms and was repeated 10 times with a pause of 1 s between each iteration.

### 3.4 End of experiment

1. Stop data recording
2. Save data and close IOX2 and MC Stimulus II software programs
3. Remove the ECG needle electrodes
4. Euthanize the mouse by an intraperitoneal injection of 100 μL of Euthasol
5. Release the distal suture to carefully remove the catheter without touching its electrodes
6. Clean the catheter with alcohol after each experiment and with Tergazyme (enzyme-active detergent) once a week

### 3.5 Data analysis

#### 3.5.1 Baseline ECG analysis

Baseline ECG parameters (heart rate, P-wave amplitude and duration, PR, QRS and QT intervals) are analyzed using the ecgAUTO software. After filtering out the 50 Hz signal, the software runs an automated morphological comparison with a library of reference complexes created by the user for each ECG recording. This automated analysis is then verified visually by the user, who is kept blind to the mouse’s genotype.

#### 3.5.2 Arrhythmia analysis

VAs induced during PES are analyzed manually using ecgAUTO software. For each protocol, all arrhythmic events are counted and sorted by type: premature ventricular complex (PVC), doublet or ventricular tachycardia (VT), i.e., ≥ 3 consecutive PVCs.

In order to quantify the severity of arrhythmic events induced by PES, we have calculated for each animal of this study and for each stimulation protocol, a score as established by Clasen et al in 2018 (Clasen et al., 2018). This score assigns 1 point for one isolated PVC, 3 points for a couplet, 4 points for a VT of 3 consecutive PVCs, 5 points for a VT of at least 5 PVCs and 6 points for a VT lasting more than 1 s. Since none of the mice in our study triggered VT approaching a duration of 1 s, we adapted the Clasen’s score by assigning 4 points to VTs of 3 consecutive PVCs and 5 points to VTs of more than 4 complexes. For each mouse, a cumulative arrhythmia score (CAS) is calculated by adding the score calculated for each stimulation protocol (S2 to S5).

## 4 Results

### 4.1 Aggressive stimulation protocols frequently trigger arrhythmias in WT mice

**FIGURE 5:**
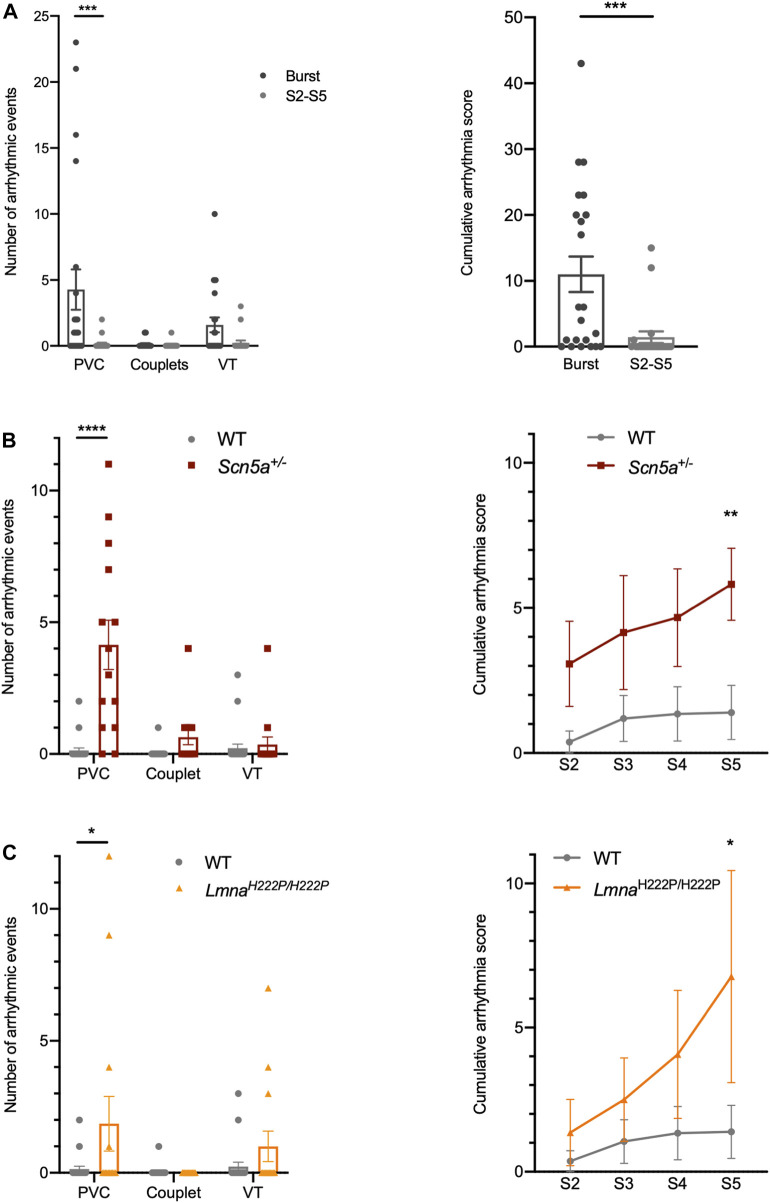
Ventricular arrhythmia induction in 2 murine models of arrhythmogenic cardiac diseases and comparison with controls. **(A)** Left: Number of arrhythmic events (PVC, couplet or VT) recorded per mouse in WT mice using either aggressive “burst” protocol (*n* = 22) or S2–S5 progressive protocol (*n* = 21). Right: Arrhythmia score calculated in WT mice using either aggressive “burst” protocol (*n* = 22) or S2–S5 progressive protocol (*n* = 21). The Mann-Whitney test was used to determine statistical significance for comparison of two groups and 2-way ANOVA for comparison of more than 2 groups. ***p < 0.001. **(B)** Left: Number of arrhythmic events (PVC, couplet or VT) recorded per mouse in WT (*n* = 21) and *Scn5a* ^+/−^ (*n* = 14) mice using S2–S5 progressive protocol. Right: Arrhythmia score calculated in WT (*n* = 21) and *Scn5a* ^+/−^ (*n* = 14) mice using S2–S5 progressive protocol. The Kruskal-Wallis test (with Dunn’s correction) was used to determine statistical significance. *p < 0.05; ****p < 0.0001. **(C)** Left: Number of arrhythmic events (PVC, couplet or VT) recorded per mouse in WT (*n* = 21) and *Lmna* ^H222P/H222P^ (n = 14) mice using S2–S5 progressive protocol. Right: Arrhythmia score calculated in WT (*n* = 21) and *Lmna* ^H222P/H222P^ (*n* = 14) mice using S2–S5 progressive protocol. The Kruskal-Wallis test (with Dunn’s correction) was used to determine statistical significance. *p < 0.05.

As said earlier in this manuscript, 2 intracardiac stimulation protocols were recently detailed (Favere et al., 2022; Hennis et al., 2022), both of them aiming at triggering arrhythmias in WT mice through aggressive “burst” stimulation programs, while we propose here more progressive protocols designed to be less aggressive. When both approaches of PES were applied to WT mice, they experienced more arrhythmic events when stimulated using “burst” protocols than when progressive protocols were applied (Table 1; Figure 5A left panel). Consequently, the CAS was significantly higher in mice stimulated by “burst” protocol than in mice stimulated by the S2–S5 protocol (Table 1; Figure 5A right panel).

**TABLE 1:** PES results obtained in WT mice and *Scn5a* ^+/−^ and *Lmna* ^H222P/H222P^ mouse models.

	WT			*Scn5a* ^+/−^		*Lmna* ^H222P/H222P^	
	“burst” *n* = 22	S2–S5 *n* = 21	*P*	S2–S5 *n* = 14	*P*	S2–S5 *n* = 14	*P*
PVC	4.3 ± 1.5	0.1 ± 0.1	^‡‡‡^	4.1 ± 0.9	****	1.9 ± 1	*
Couplet	0.1 ± 0.1	0.1 ± 0.1	ns	0.6 ± 0.3	ns	0	ns
VT	1.6 ± 0.6	0.2 ± 0.2	ns	0.4 ± 0.3	ns	1 ± 0.6	ns
CAS	11 ± 2.7	1.4 ± 0.9	^‡‡‡^	7.5 ± 1.9	***	6.4 ± 3.4	ns

^‡‡‡^ *p* < 0.001; ns = not significant when compared to WT burst values. *p < 0.05; ***p < 0.001; ****p < 0.0001; ns = not significant when compared to WT S2–S5 values. CAS, cumulative arrhythmia score.

These results suggest that the progressive PES protocols proposed may be less aggressive than “burst” programs and are more suitable for revealing arrhythmia susceptibility in pathological models. Indeed, if we consider that normal mice should not have a substrate enabling arrhythmias to be induced by PES protocols, then the protocols proposed here may be more specific about the presence of an arrhythmogenic substrate and would enable us to better differentiate between the normal and the pathological.

### 4.2 Progressive S2–S5 stimulation protocols demonstrated higher susceptibility to arrhythmias in *Scn5a* ^+/−^ and *Lmna* ^H222P/H222P^ models, when compared to controls

To induce VAs in a Brugada-like model (*Scn5a* ^+/−^) and in a model of arrhythmogenic cardiomyopathy (*Lmna* ^H222P/H222P^), we chose to apply progressive protocols with the aim to assess the susceptibility of these models to PES-triggered arrhythmias by comparison with WT mice. Table 1 and Figure 5B show that *Scn5a* ^+/−^ mice triggered more arrhythmic events than WT mice and had a higher score than control mice at each step of the protocol (S2: 3.1 ± 1.5 vs*.* 0.4 ± 0.4; S3: 4.2 ± 2 vs*.* 1.2 ± 0.8; S4: 4.7 ± 1.7 vs*.* 1.4 ± 0.9; S5: 5.6 ± 1.2 vs*.* 1.4 ± 0.9, *p* < 0.05 in *Scn5a* ^+/−^ (*n* = 14) vs*.* WT (*n* = 21) mice, respectively) and a significantly higher CAS than WT mice. As shown in Table 1 and Figure 5C, *Lmna* ^H222P/H222P^ mice triggered more arrhythmic events than WT mice and had a higher score than control mice at each step of the protocol (S2: 1.4 ± 1.1 vs*.* 0.4 ± 0.4; S3: 2.5 ± 1.4 vs*.* 1.2 ± 0.8; S4: 4.1 ± 2.2 vs*.* 1.4 ± 0.9; S5: 6.9 ± 3.7 vs*.* 1.4 ± 0.9, *p* < 0.05 in *Lmna* ^H222P/H222P^ (*n* = 14) vs*.* WT (*n* = 21) mice, respectively). While higher in *Lmna* ^H222P/H222P^ than in WT mice, the CAS was not significantly different in both groups. Altogether, these results suggest that both Brugada-like and arrhythmogenic-cardiomyopathy murine models are more susceptible to trigger arrhythmic events than control mice when subjected to progressive S2–S5 protocols.

## 5 Discussion

The aim of this study was to provide the research community with ready-to-use and standardized protocols to perform PES in mice for characterizing arrhythmias in transgenic murine models of cardiac diseases without any pharmacological provocation. We described the surgical procedure to perform PES in mice, detailed all needed materials and we proposed novel protocols, based on the repetition of 2–4 extrastimuli, adapted from human clinical practice. In addition to this methodological description, we demonstrated that these protocols enabled us to characterize 2 models of channelopathy and arrhythmogenic cardiomyopathy, highlighting their greater susceptibility to PES-triggered arrhythmias, when compared with control mice.

### 5.1 Progressive PES protocols revealed greater susceptibility to arrhythmias in both disease models

The “burst” protocol has been widely used to realize PES for its effectiveness in triggering arrhythmic events *in vivo* in mice and *ex vivo* on explanted hearts (Ripplinger et al., 2022). However, this approach efficiency is at the expense of its specificity, since many WT mice trigger arrhythmic events with this protocol. In our experience, 73% of WT mice triggered at least one event when stimulated by the “burst” protocol compared to 19% when stimulated with the S2–S5 progressive protocol (Figure 5A). The number of PVCs was significantly higher in “burst”-simulated mice than in the S2–S5-stimulated group, and the number of VTs was also increased by the stimulation protocol aggressiveness. As a result, the cumulative score calculated in the “burst” stimulated group of mice was significantly higher than the score calculated in S2–S5 stimulated mice.

It is worth to note that we chose to define VT as a repetition of 3 consecutive PVCs as in human clinics (Zeppenfeld et al., 2022) and unlike other studies considering VT as a repetition of at least 4 consecutive PVCs (Curtis et al., 2013). This could explained why we observed 2 WT mice triggering VTs while stimulated with the non-aggressive S2–S5 protocols. In their initial scoring system, Clasen *et al.* assigned more points to a VT lasting more than 1 s than to a VT of less than 1 s to emphasize the severity of a long VT event (Clasen et al., 2018). Since none of the mice in our study triggered VT approaching a duration of 1 s, we adapted the Clasen’s score by assigning 4 points to VTs of 3 consecutive PVCs and 5 points to VTs of more than 4 complexes.

Although mice are unlikely to spontaneously present arrhythmias (Nerbonne, 2014), we believe that it is not relevant to apply aggressive PES protocols as the results obtained might not be specific and include many false positives (Maguire et al., 2003). In this respect, our study is interesting in that it proposes protocols derived from human practice which, by bringing extrastimuli close to the refractory period of the stimulated ventricular area, favor reentry phenomenon and enable to challenge disease hearts and to specifically induce arrhythmic events. When applied to the *Scn5a* ^+/−^ or the *Lmna* ^H222P/H222P^ transgenic mice models, S2–S5 progressive protocols revealed an increased susceptibility to arrhythmias triggered by intracardiac stimulation, when compared to WT mice (Table 1; Figures 5B, C). However, this increase was statistically significant only for the *Scn5a* ^+/−^ group. These observations are consistent with the intrinsic characteristics of each model, since the *Scn5a* ^+/−^ line is a model of channelopathy (arrhythmia only) whereas the *Lmna* ^H222P/H222P^ line is a model of cardiomyopathy which seems more arrhythmic than WT mice but less than *Scn5a* ^+/−^ mice.

### 5.2 Advantages and disadvantages

Several techniques can be used to induce and record ventricular arrhythmias in mice such as telemetry and optical mapping (Ripplinger et al., 2022). The technique of endocavitary PES through the external jugular vein described in this study offers several advantages. It reproduces a methodology used in clinical practice to induce ventricular arrhythmias in patients for diagnostic and/or prognostic purposes (Sroubek et al., 2016; Zeppenfeld et al., 2022), based on the measurement of ventricular refractory periods, enabling pacing cycles to be adapted to each mouse and avoiding overly aggressive pacing. Distinguishing the normal from the pathological in humans, like the WT from the transgenic strains in mice, requires adapted and progressive protocols. Indeed, as in clinical practice, the use of aggressive protocols allows the induction of ventricular arrhythmias in both healthy and mutated mice, blurring the differences and therefore the conclusions. In fact, like other teams (Clasen et al., 2018), we have shown a marked increase in the occurrence of ventricular arrhythmias with an aggressive “burst”-type protocol in control mice. That said, “burst” protocols induce a calcium overload capable of triggering focal arrhythmias, which may make them interesting for studying some pathological models, whereas progressive protocols, such as S2–S5, were designed to trigger reentrant arrhythmias of the type seen in Brugada patients. It should also be noted that, to limit the potential intracellular calcium overload linked to the repetition of stimulations that could lead to arrhythmias, a pause of 1 minute was systematically observed between the “burst” protocol and the progressive S2–S5 protocol. Besides, since we observed an increase of the number of arrhythmic events triggered in both models (Table 1; Figures 5B, C), we have limited PES protocols to 4 extrastimuli in order to reduce manipulation time. But the principle of this progressive PES protocol can be extended to include additional extrastimuli, depending on the model studied. It would then be important to keep in mind that from 3 to 4 extrastimuli onwards, WT mice may also trigger arrhythmic events.

This technique enables us to work on living animals without major surgery, unlike thoracotomy for epicardial pacing (Berul et al., 1996) or laparotomy for subdiaphragmatic pacing (Gutstein et al., 2003) performed by other teams. It does not dispense with general anesthesia and its antiarrhythmic effect by depressing adrenergic tone (Chaves et al., 2003). It does, however, allow a reduction in anesthetic doses and the maintenance of spontaneous ventilation, all of which are conducive to the maintenance of relatively physiological loading conditions, limiting the hemodynamic impact of these manipulations on mice with advanced left ventricular dysfunction.

Another advantage of this technique is its capacity to highlight atrial and atrioventricular conduction capacities. By positioning the distal electrode in the right atrium, intracardiac catheterization via the jugular vein allows further electrophysiological characterization: sinus node recovery time, atrial effective refractory period, atrioventricular effective refractory period and Wenckebach periodicity (Mangoni et al., 2006). PES technique enables electrophysiological exploration of the entire heart in the same experiment. As mentioned above, we have chosen to detail here only ventricular stimulation protocols adapted to the 2 disease-models studied since atrial stimulation protocols were detailed elsewhere (Favere et al., 2022).

The use of an arrhythmia score is encouraged to characterize a model and quantify data according to the guidelines for the study of animal and human ventricular and supraventricular arrhythmias (Curtis et al., 2013). The results relating to raw arrhythmia data remain the most important (Curtis and Walker, 1988) and certain limitations are linked to the counting of events within the score. Indeed, the score does not take into account the polymorphic nature of arrhythmias, which may reflect more severe ventricular tachycardias or more complex mechanisms. As arrhythmias generally take a few beats to become monomorphic, and arrhythmias observed in this study are rarely sustained over time, the relevance of taking morphology into account is limited. Besides, if the cumulative arrhythmia score assigned to a mouse should not be considered as a linear marker of arrhythmogenicity, it is much more powerful than a simple threshold, since it weights arrhythmic events according to their severity (PVS vs*.* VT) and repetition in the same animal. In conclusion, calculating scores allows to avoid a binary definition - yes or no - of arrhythmia, thus complementing the raw data without replacing it.

### 5.3 Limitations of intracardiac PES

Despite the increasingly widespread use of PES in mice, several challenges and limitations persist. The main limitation of this technique is that it is invasive and terminal by nature and thus, does not authorize repeated measures in the same animal. Moreover, intracardiac PES is a time-consuming experiment requiring a learning curve that may take several weeks. Also, the general anesthesia of the mouse may alter its susceptibility to arrhythmic events and restrict the maximum number of additional extrastimuli that can be applied within the experiment duration. Finally, limitations have to be considered when extrapolating experimental data from small animals to patients.

## Data Availability

The original contributions presented in the study are included in the article/supplementary materials, further inquiries can be directed to the corresponding author.
